# Dementia and Mental Health During the COVID-19 Pandemic: A Systematic Review

**DOI:** 10.3389/fpsyt.2022.879598

**Published:** 2022-07-07

**Authors:** Julia Mariano Gaigher, Isabel Barbeito Lacerda, Marcia Cristina Nascimento Dourado

**Affiliations:** Center for Alzheimer's Disease, Institute of Psychiatry, Universidade Federal Do Rio de Janeiro, Rio de Janeiro, Brazil

**Keywords:** COVID-19, dementia, anxiety, mental health, depression, caregiver

## Abstract

**Objectives:**

The COVID-19 pandemic raised significant concerns related to the management of care for people with dementia, but few studies have examined the mental health of older adults with dementia and their caregivers during the pandemic, when compared to other populations. This systematic review thus aims to compare and discuss the impact of the COVID-19 pandemic on people with dementia and on their caregivers' mental health.

**Methods:**

A search was performed in the PubMed/Medline and ISI databases according to the PRISMA methodology. We included studies published in 2020 and 2021 with the following combinations of keywords: “COVID-19 and mental health and elderly,” “COVID-19 and mental health and dementia;” “COVID-19 and dementia and caregivers,” “pandemic and mental health and elderly,” and “pandemic and anxiety.”

**Results:**

Twenty-two studies were included. Technology has proven to be an essential ally during the pandemic, since all 22 studies performed remote data collection. Nearly all the studies emphasized that social isolation and withdrawal can lead to the emergence or increase of neuropsychiatric symptoms and motor difficulties. However, the findings were mixed concerning the pandemic's impact on the cognition of people with dementia. Caregivers also suffered from the pandemic's impact, experiencing an increase in the burden of care and symptoms of stress, depression, and anxiety. Few studies suggested measures to alleviate the difficulties of people with dementia and their caregivers. There are reports of the benefits of technology in communication and treatment through teleconsultations, however, not everyone has access to such technology, making it difficult to disseminate this tool to the target population.

**Conclusions:**

The studies generally showed that social isolation can increase motor deficits and neuropsychiatric symptoms and caregivers' burden and anxiety. New avenues for care and intervention are thus needed for older adults with cognitive deficits and their caregivers to avoid the intensification of physical and psychological suffering. Technological initiatives and support should consider people with cognitive impairment and different levels of technology literacy.

**Systematic Review Registration:**

https://www.crd.york.ac.uk/prospero/.

## Introduction

In late 2019, the novel coronavirus SARS-CoV-2 was identified as the cause of COVID-19, a respiratory disease with varying individual severity. In March 2020, as the disease was spreading worldwide, the World Health Organization (WHO) declared COVID-19 a pandemic. Research and experience have shown that COVID-19 severity and case-fatality are associated with the individual's age and immune status (1). Older age is thus considered a risk factor. The mortality rate is higher in the elderly due to several characteristics such as comorbidities, lower antibody levels, and polypharmacy, among others (2). Various governmental guidelines on COVID-19 have thus focused on older adults (3).

Social isolation is a useful measure for controlling the spread of infectious diseases or protecting high-risk groups from negative health outcomes. However, social isolation can also result in sedentary behavior, which is detrimental to the prevention of physical, psychological, and social health problems (4). In older adults, social isolation can increase the risk of depression, anxiety, and suicide, with considerable impact on quality of life, burden of care, and use of resources. For example, Rana (5) described five reported cases of older adults who committed suicide due to recurrent depressive disorder. Older adults already suffering from mental disorders have been more vulnerable to COVID-19 and its social consequences (5).

Social isolation is difficult for people with dementia and their caregivers in this context. According to Dourado et al. (6), COVID-19 raised significant concerns in the management of care for people with dementia. This age group has experienced limited access to services and activities, resulting in aggravation of cognitive deficits, affecting such domains such as memory and orientation, besides behavioral impairments. Social isolation can also exacerbate preexisting stress, overburden, and depression in caregivers (6).

The pandemic has further aggravated the vulnerability of older adults, especially those with neurocognitive disorders such as Alzheimer's disease. For example, dementia can increase the risk of contracting COVID-19, due to difficulties in understanding or remembering the need for social isolation (6). The COVID-19 pandemic also involves caring for people with dementia and support from community centers for this patient population, when such centers are experiencing difficulties continuing their work (6). The main objective of this systematic review was thus to better understand the impacts of the COVID-19 pandemic on people with dementia and on their caregivers' mental health.

## Methods

This systematic review was performed according to the Preferred Reporting Items for Systematic Reviews and Meta-Analyses (PRISMA) (7). The literature search was carried out from August 5 to 26, 2021, using the following electronic databases: Medline (PubMed) and Science Citation Index (Institute for Scientific Information – ISI). Based on Medical Subject Headings (Mesh), the search keywords included “COVID-19,” “pandemic,” “mental health,” “dementia,” “caregivers,” and “elderly” in the following combinations: “COVID-19 and mental health and elderly,” “COVID-19 and mental health and dementia,” “COVID-19 and dementia and caregivers,” “pandemic and mental health and elderly,” and “pandemic and anxiety.”

The search was performed according to the following PICOS:

Population: older adults
Intervention: COVID-19; social isolation
Control: older adults with dementia; caregivers of people with dementia
Outcome: mental health, stress, depression, anxiety, neuropsychiatric symptoms, cognition
Study design: a review of cross-sectional, longitudinal, randomized, nonrandomized, and case-control studies.

Inclusion criteria were: (1) publications from 2020 to 2021, (2) only studies with older adults with cognitive impairment and/or their caregivers, (3) research on people with dementia (cognition, neuropsychiatric symptoms, and functionality) and their caregivers' mental health (burden, anxiety, and depression) during the COVID-19 pandemic, and (4) publications in the English language. The exclusion criteria were: (1) studies published prior to 2020 and (2) mental health studies during the COVID-19 pandemic without people with dementia and/or their caregivers.

### Study Selection

#### First Step

Two independent reviewers performed initial article screening by reading the titles and abstracts. Reviewers excluded articles that failed to meet the eligibility criteria and retained those that were possibly eligible. In cases where there was no clear consensus between the reviewers, the article remained among those potentially eligible and moved on to the next phase of eligibility assessment. A third independent reviewer (IL) resolved disagreements between reviewers.

#### Second Step

The full texts of articles selected in the first phase were read by two independent reviewers to verify eligibility. In this phase, the primary reasons for excluding articles were recorded in the PRISMA article selection flowchart.

#### Third Step

All selected articles were submitted to the Mixed Methods Appraisal Tool (MMAT), version 2018 (8), a critical quality appraisal tool for scientific studies. The MMAT establishes corresponding criteria for each research method, and scores are rated from one to five, considering the description of each stage of the method's implementation.

This systematic review was recorded in the International Prospective Register of Systematic Reviews (PROSPERO), CRD42021276339.

## Results

**Figure 1:**
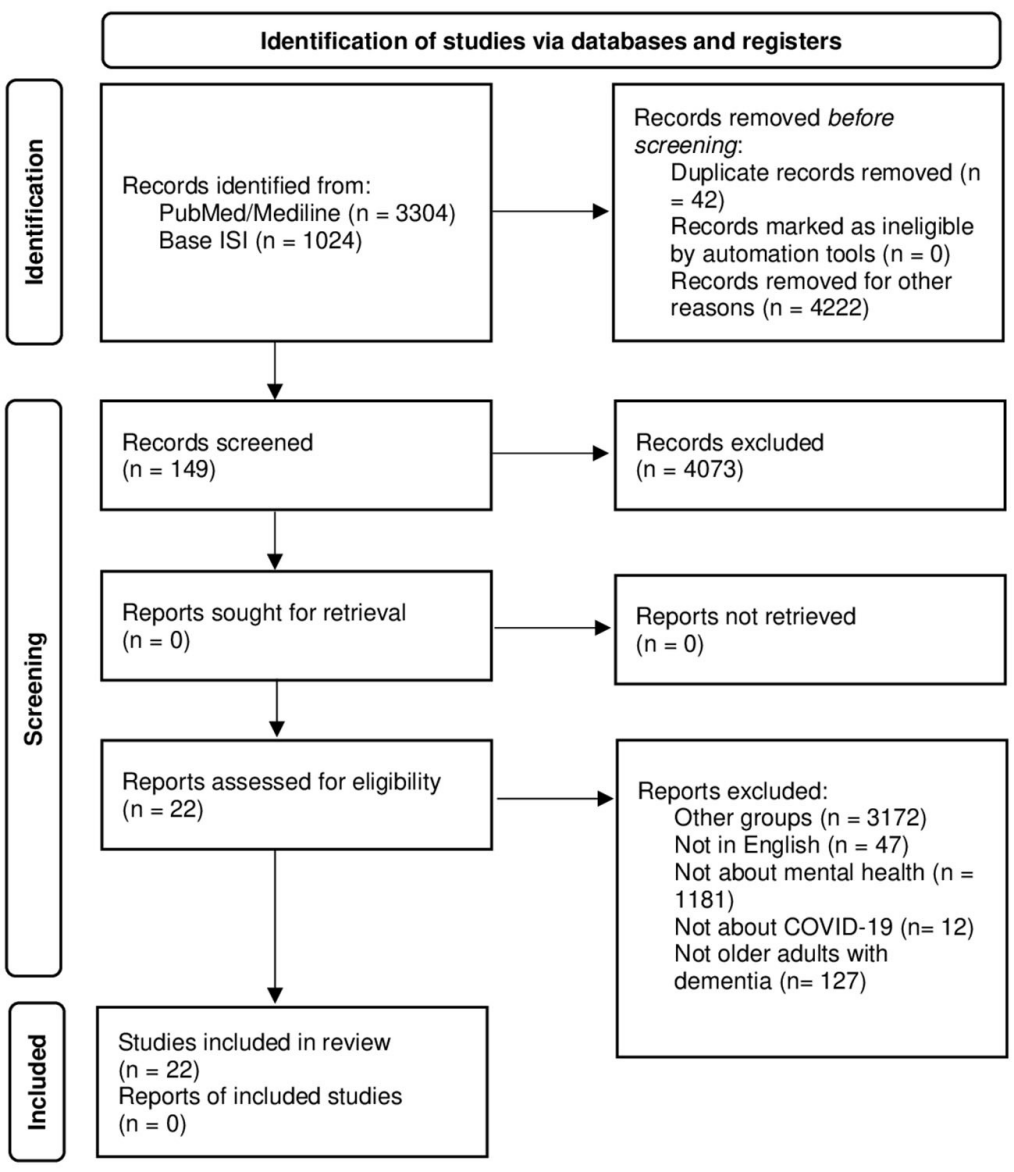
Flow chart describing data extraction.

Initially, 4,328 records were identified through the database searches: 3,304 in PubMed/Medline and 1,024 in ISI. The 149 studies that remained after application of the exclusion criteria were retrieved for potential use, and the information of the full-text version of each study was evaluated. The reference lists of all selected articles were cross-referenced. After duplicates were removed, the total number of studies decreased to 22. Figure 1 provides a flowchart of the different study selection phases. The included studies are shown in Table 1.

**Table 1:** Selected studies.

**Author/year**	**Country/ study design/** **participants**	**Objective**	**Results**	**Quality** **assessment**
El Otmani et al. (22)	Morocco/ prospective/50 people with PD	Determine the impacts of the pandemic on depression and anxiety in people with PD.	After 6 weeks of confinement, there was no statistically significant difference in either depression or anxiety compared to the first evaluation.	****
Gan et al. (11)	China/ retrospective descriptive/205 elderly people with cognitive impairment	Investigate cognitive and neuropsychological changes as well as proportions of rapid cognitive decline before and during the COVID-19 pandemic.	There was no significant difference in dementia severity scores or the proportions of neuropsychiatric symptoms between the initial and final evaluations. The scores on the C-MMSE, MoCA, ADLs, and global NPI differed significantly between baseline and follow-up evaluations after almost 14 months.	****
Giebel et al. (12)	United Kingdom/ Longitudinal/223 older adults, 285 caregivers and 61 PLWD	Explore how social support services and mental well-being for older adults, carers, and people with dementia changed over the first 3 months since the start of the pandemic.	Social support service usage dropped shortly after lockdown measures were imposed at T1, then increased again by T3. Access to paid care was least affected by COVID-19. Cases of anxiety dropped significantly across the study period, while cases of depression increased. Well-being increased significantly for older adults and PLWD from T1 to T3.	*****
Hanna et al. (28)	United Kingdom/ Qualitative/ 15 unpaid caregivers, 1 ex-caregiver, and 4 PLWD	Explore the change in impact of COVID-19 public health measures on the mental wellbeing of people with dementia and unpaid caregivers.	Loss of social support services was key in impacting this cohort mentally and emotionally, revealing the need for better psychological support for both caregivers and PLWD.	*****
Morii et al. (24)	Japan/ Cross-sectional/ 88 patients and their family members, 44 with Parkinson's disease, and 44 controls	Investigate the impact of social restrictions during the COVID-19 pandemic on neuropsychiatric symptoms in PD patients and identify risk factors associated with these symptoms.	PD patients may be more likely to develop clinical depression than those without PD in the presence of social stressors such as a pandemic, even in Japan where no legal penalties were imposed during the state of emergency.	*****
Manini et al. (20)	Italy/ cross-sectional/ 94 elderly people with dementia	Assess the impact of prolonged lockdown on behavioral and psychological symptoms of dementia.	Mean total NPI score before March 9 was 9.0 (SD 5.0), whereas the caregiver distress scale showed a mean score of 4.5 (SD 3.0). Scores increased respectively to 11.5 (9.0) and 5.5 (5.0) during nationwide lockdown.	****
Portacolone et al. (15)	USA/ Qualitative/ Adults aged 55 and older with cognitive impairment, living alone	Assess how older adults with cognitive impairment are coping with the pandemic.	The pandemic highlighted the precarity and unmet needs of older adults with cognitive impairment living alone. Findings underscore the need to expand access to home care and mental health services for this population.	*****
Tsapanou et al. (16)	Greece/ Cross-sectional/ 204 caregivers, 36 MCI, 58 all-stage dementia.	Analyze the impact of COVID-19 pandemic on older adults with MCI/dementia and their caregivers.	There was a significant overall decline for people with MCI/dementia in communication, mood, movement, and compliance with lockdown measures. Caregivers showed a major increase in their psychological and physical burden.	****
Altieri et al. (27)	Italy/ Cross-sectional/ 84 caregivers of people with dementia	Assess the psychological impact of the pandemic and COVID-19 social isolation on caregivers of people with dementia.	Multivariate analysis of variance revealed an effect of time (before and during lockdown) in the whole group on depression scores; a significant interaction between time and resilience was found on anxiety scores, where caregivers with high resilience showed a more significant increase in anxiety levels during lockdown than caregivers with low resilience. Caregiver burden was associated negatively with resilience scores and positively with higher functional dependence.	****
Barguilla et al. (9)	France/ Cross-sectional/ 60 people mild cognitive decline and dementia	Describe the influence of restrictive measures on patients with mild cognitive decline and dementia evaluating SARS-CoV-2 infection, changes in routines, cognitive decline, neuropsychiatric symptoms, delirium, falls, caregiver stress, and access to healthcare.	70% of patients abandoned previous daily activities, 60% had cognitive decline reported by relatives/caregivers, 15% presented delirium episodes, and 13% suffered increased incidence of falls. Caregivers reported increased burden in 41% of cases and burnout in 11% of cases. 16% reported difficulties accessing medical care, 33% received medical assistance via telephone, 20% needed emergency care, and 21% had changes in psychopharmacological therapies.	****
Boutoleau-Bretonnière et al. (17)	France/ Cross-sectional / 38 participants with clinical diagnosis of probable AD	Investigate the effects of confinement during COVID-19 on neuropsychiatric symptoms in patients with AD.	Only ten of 38 patients showed neuropsychiatric changes during confinement. Cognitive function of these ten patients, assessed with the Mini-Mental State Examination, was worse than that of patients who did not show neuropsychiatric changes. Duration of confinement correlated significantly with severity of symptoms as well as with their caregivers' distress.	****
Boutoleau-Bretonnière et al. (21)	France/ Cross-sectional/ 78 caregivers of people with bvFTD and AD	Investigate the impact of home confinement during COVID-19 on the burden of caregivers of bvFTD or AD patients.	22 bvFTD caregivers and 14 AD caregivers experienced an increase in burden. For bvFTD caregivers, this increased burden occurred regardless of behavioral changes, while AD caregivers experienced increased burden related to changes in patients' neuropsychiatric symptoms.	****
Cohen et al. (18)	Argentina/ Cross-sectional/ 119 individuals with AD and related dementia and their families	Study to what extent social isolation affected behavioral symptoms in persons with dementia after the first 8 weeks of quarantine.	Symptoms of anxiety, depression, and sleep disorders were reported in 33, 12.8, and 14.7% of the sample, respectively. New onset of behavioral symptoms or exacerbation of preexisting behavioral symptoms showed positive correlation with patient age and with presence of anxiety reported before	*****
			the epidemic and negative correlation with the global CDR score and the memory, community affairs, and home and hobby domains of CDR.	
Di Santo et al. (10)	Italy/ Cross-sectional/ 126 community-dwelling seniors with MCI or SCD	Explore the effects of COVID-19 and quarantine measures on the lifestyles and mental health of older adults at increased risk of dementia.	Over 1/3 of the sample reduced their physical activity and nearly 70% reported an increase in idle time. Adherence to the Mediterranean diet decreased in almost 1/3 of respondents, and over 35% reported weight gain. Social activities were abolished and 1/6 of participants also decreased productive and mentally stimulating activities. There was a significant association between depression and living alone or having poor relations with cohabitants and between anxiety and SCD, cold or flu symptoms, and reduction in productive and leisure-time activities.	*****
El Haj et al. (19)	France/ Cross-sectional/ 58 participants with clinical diagnosis of probable AD	Investigate the effects of measures against COVID-19 on the mental health of people with AD living in nursing homes.	Participants reported higher levels of depression and anxiety during the COVID-19 pandemic compared to data collected before the pandemic.	****
Goodman-Casanova et al. (13)	Spain/ Cross-sectional/ 93 people with MCI or mild dementia and their caregivers	Explore the impact of confinement on the health and well-being of community-dwelling older adults with MCI or mild dementia.	Health status was found to be optimal in 96% of respondents with no COVID-19 symptoms. Participants living alone reported greater negative feelings and more sleep problems. Concerning leisure-time activities, 57% respondents took walks, 35% played memory games, 60% watched TV, and 98% telephoned relatives.	****
Janiri et al. (23)	Rome/ Cross-sectional/ 134 individuals with Parkinson's disease	Identifying risk/protective factors associated with subjective worsening of psychiatric symptoms during COVID-19 in a sample of individuals with PD 65 years or older.	101 participants reported lifetime psychiatric symptoms. Among these, 23 displayed subjective worsening of psychiatric symptoms. In this group, the most frequent symptom was depression (82.6%), followed by insomnia (52.2%). Subjective worsening of neurological symptoms and lifetime irritability, together with younger age and female sex, were specific risk factors for worsening of psychiatric presentation. Lifetime preexisting delusions, having received antipsychotics, and not having received mood stabilizers were also associated with subjective worsening of psychiatric symptoms during the COVID-19 pandemic.	****
Lai et al. (29)	China/ Cross-sectional/ 60 caregivers of people with dementia	Evaluate whether supplementary telehealth via video-conferencing platforms could bring additional benefits for individuals with NCD and their spousal caregivers at home.	Supplementary telemedicine averted deterioration in the Montreal Cognitive Assessment, evident in the telephone-only group. It also reversed the downward trend in quality of life observed in the telephone-only group. Varying degrees of improvements in physical and mental health, perceived burden, and self-efficacy were observed among caregivers in the video-conferencing group, which were absent in the telephone-only group.	****
Lara et al. (14)	Spain/ Cross-sectional/ 40 people diagnosed with MCI or mild AD	Analyze the pandemic's impact on the neuropsychiatric symptoms of people with AD and MCI and their quality of life after a 5-week lockdown.	There was worsening in NPI scores after confinement *(P* = 0.028). The most frequently affected neuropsychiatric symptoms were apathy and anxiety in patients with MCI and apathy, agitation, and aberrant motor behavior in patients with AD. No differences were seen in quality-of-life scores during the re-evaluation. 30% of patients and 40% of caregivers reported worsening of patients' health status during confinement.	****
Prasad et al. (25)	India/ Cross-sectional/ 100 people with PD and their caregivers	Explore the effects of prolonged lockdown on people with PD.	There was a significant increase in inability to access health care and difficulty in obtaining medication. Patients also reported worsening of motor symptoms.	****
Vaitheswaran et al. (30)	India/ Qualitative/ 31 caregivers of people with dementia	Describe the experiences and needs of caregivers of persons with dementia during the COVID-19 pandemic and lockdown in a city in India.	Thematic data analysis showed two sets of issues that caregivers of persons with dementia experienced during the pandemic. The first was unique to caregivers directly related to their caregiving role, while the second was not related directly to their caregiving role. These two sets also appeared to display two-way interaction. These issues generated needs, some of which required immediate support while others required long-term support. Caregivers suggested several methods such as video-consultations, telephone-based support. and clinic-based in-person visits to meet their needs. They also wanted more post pandemic services.	*****
Xia et al. (26)	China/ Cross-sectional/ 119 Chinese with PD	Investigate the incidence of anxiety, depression, and sleep disorders in PD patients and compare to controls to determine the impact of PD on mental and sleep states.	Compared to healthy controls, sleep disorders were identified in 68.9% of PD patients. Sleep disorder was independently associated with exacerbation of PD symptoms and anxiety. Compared to male PD patients, female patients had higher PSQI scores as well as anxiety and depression prevalence.	****

PD, Parkinson's disease; PLWD, people living with dementia; MCI, mild cognitive impairment; AD, Alzheimer's disease; bvFTD, behavioral variant frontotemporal dementia; SCD, subjective cognitive decline; NCD, neurocognitive disorder. Quality assessment: ^*^lower quality to ^*****^higher quality.

### Participants

Some studies were only carried out in individuals with cognitive impairment (9–16). The types of dementia in the studies included Alzheimer's disease (9, 11, 14, 16–21), mixed dementia (20), vascular dementia (20), Lewy body dementia (20), Parkinson's disease (22–26), and frontotemporal dementia (20). Several studies focused only on caregivers' health and burden (12, 13, 16–18, 27–30).

### Study Designs

Most of the studies used quantitative designs: one prospective study (22), one retrospective descriptive study (11), one longitudinal survey (12) and 16 cross-sectional studies (9, 10, 13–21, 23–30). There were also three qualitative studies (15, 28, 30). Based on the MMAT criteria, eight studies were classified as displaying high methodological quality (10, 12, 15, 18, 24, 28, 30).

Several cross-sectional studies (16, 17, 20, 21, 29) used caregivers' reports to evaluate changes before and during the pandemic. One study compared people with Parkinson's disease to controls (24). Some studies (18, 27) used online surveys targeted to groups dedicated to people with dementia and/or caregivers, online newspapers, and caregivers' associations. A single study (10) included community-dwelling seniors enrolled in a suspended randomized controlled trial. Some studies (14, 25, 26) included participants that had a previous evaluation as a normal procedure included in their unit. One study (23) consecutively enrolled participants who had a scheduled medical visit during COVID-19 lockdown. Another study (9) used data from databases and previous research from laboratories and clinics to assess measures before social isolation; during the pandemic, the same patients already belonging to the database were reassessed.

### Assessments

All the selected studies followed recommendations from health authorities, so that the assessments were done remotely by phone calls or with an online form and video calls. Some studies used data stored in databases to compare characteristics before and during the pandemic (9, 11, 12). The scales used for the online assessment of depression and anxiety were the Hospital Anxiety and Depression Scale (HADS) (19, 22, 27), Personalized Health Questionnaire 9 (PHQ-9) (12, 24) and Generalized Anxiety Disorder 7 (GAD-7) (10, 12, 24). The Geriatric Depression Scale-5-item (GDS-5) was adapted (10, 13). The Adult Resilience Scale (RSA) (27), was used in the assessment of caregivers' resilience. Sleep quality was measured online with the Insomnia Severity Index (ISI) (24). Cognitive assessment was performed with the Mini-Mental State Examination (MMSE) (10, 11, 13, 19, 20). Montreal Cognitive Assessment – MoCA (11, 29), and Clinical Dementia Rating Scale (CDR) (9, 11, 18). Neuropsychiatric symptoms were assessed with the Neuropsychiatric Inventory (NPI) (9, 11, 14, 17, 20). Functionality was assessed with the Functional Assessment Questionnaire (FAQ) (10) and Basic Activities of Daily Living (ABVD) (10, 11). Caregiver burden was assessed with the Caregiver Burden Inventory (CBI) (27) and Zarit Burden Interview Scale (ZBI) (29) Neurological characteristics were assessed with the Unified Parkinson's Disease Rating Scale (UPDRS) (23). Quality of life was measured with the Short Warwick-Edinburgh Mental Well-Being Scale (SWEMWBS) (12) and Quality of Life in Alzheimer's Disease Assessment (QoL-AD) (14).

### Cognition

The review revealed mixed results concerning the pandemic's impact on the cognition of people with dementia. Gan et al. (11) found no significant differences in dementia severity but a significant difference in the MoCA and C-MMSE scores between baseline and follow-up of people with Alzheimer's disease. Conversely, Barguilla et al. (9) identified worsening of cognitive status in 60% of people with dementia as reported by caregivers. In addition, one study found worse cognition in Alzheimer's patients with increased levels of neuropsychiatric symptoms. Boutoleau et al. (17) also reported an association between neuropsychiatric changes and cognition during COVID-19 lockdown. The cognitive function of people with dementia with increased neuropsychiatric symptoms was worse than that of those who did not show neuropsychiatric changes (17).

### Mood

Nine studies investigated changes in depression and anxiety in people with dementia (10, 12, 13, 16, 18, 19, 22–24). Most showed an increase in symptoms of anxiety and depression. In Di Santo et al. (10), the participants' scores indicated an increase in depressive symptoms during the pandemic associated with living alone or lack of good relations with others in stay-at-home isolation.

According to a longitudinal study by Giebel et al. (12), cases of anxiety decreased significantly during the study period, while cases of depression increased. Tsapanou et al. (16) reported a significant decline in communication, mood, movement, and compliance with new measures in individuals with mild cognitive impairment or dementia. Cohen et al. (18) found worsening of symptoms of depression, anxiety, and insomnia in individuals with milder stages of dementia than those in more severe stages, possibly because they were more aware of the pandemic's consequences. El Haj et al. (19) investigated the effects of COVID-19 containment measures on the mental health of people with Alzheimer's disease living in nursing homes and reported higher levels of depression and anxiety during the pandemic compared to before. Janiri et al. (23) found that subjective worsening of neurological symptoms and lifetime irritability, together with younger age and female sex, were specific risk factors for worsening of psychiatric status. Meanwhile, El Otmani et al. (22) and Kitani et al. (24) reported no significant changes in mood.

### Neuropsychiatric Symptoms

The COVID-19 pandemic and social isolation have led to significant neuropsychiatric symptoms and cognitive changes in people with dementia. Agitation, delirium, irritability, apathy, aggression, anxiety, indifference, and mood were the most common symptoms found in the studies (9, 11, 14, 17, 18, 20). Barguilla et al. (9) reported the presence of delirium in individuals with more severe stages of dementia. According to Gan et al. (11), global NPI scores differed significantly between baseline and follow-up evaluations nearly 14 months later. Lara et al. (14) reported that apathy and anxiety were the most frequent in participants with mild cognitive impairment, compared to apathy, agitation, and aberrant motor behavior in participants with Alzheimer's disease. Additionally, 30% of patients and 40% of caregivers reported worse health status of people with dementia during confinement.

Bouteleau et al. (17) found a correlation between duration of confinement and severity of neuropsychiatric symptoms. One study (18) reported that neuropsychiatric symptoms were more frequent in individuals with mild dementia compared to advanced dementia. In addition, new onset of behavioral symptoms or exacerbation of preexisting behavioral symptoms were positively correlated with patient's age and presence of anxiety before the pandemic and negatively correlated with the global Clinical Dementia Rating scores and the domains of memory, community affairs, and home and hobbies (18).

Several studies specifically focused on persons with Parkinson's disease (22–26). Deterioration in motor performance was the most prominent deficit, with evident worsening of slowness, followed by depression. There was also a decrease in sleep quality, with a reduction in sleep time and the need for sleep medication. The results also suggested that poor sleep was significantly associated with postural instability and gait disturbance. Sleep disturbances in people with Parkinson's disease can exacerbate disease symptoms, anxiety, and depression (26).

El Otmani et al. (22) found no difference in anxiety and depression in people with Parkinson's disease. Conversely, Janiri et al. (23) reported depression in 26% of Parkinson's disease patients. Preexisting lifetime delusions, having received antipsychotics, and not having received mood stabilizers were also associated with subjective worsening of psychiatric symptoms during the COVID-19 pandemic (23). Morii et al. (24) found that Parkinson's disease patients were more likely to develop clinical depression than those without the presence of social stress, even in Japan where no legal penalties were imposed for failure to comply with social isolation. Prasad et al. (25) reported a significant increase in the inability to access healthcare and difficulty in obtaining medication.

### Functionality

Barguilla et al. (9) evaluated changes in the routines of people with dementia and found that 70% of participants abandoned previous daily activities and 13% suffered increased incidence of falls. In Di Santo et al. (10), more than one-third of the sample reduced their physical activity and eliminated their social activities, one-sixth also decreased their productive and mentally stimulating activities, and nearly 70% reported an increase in idle time. Interestingly, according to Goodman-Casanova et al. (13), in Spain, health status was found to be optimal in 96% of respondents with no COVID-19 symptoms, 35% played memory games, 60% watched television, 98% telephoned relatives, and 57% of those with mild cognitive impairment or mild dementia took walks. (13) Lara et al. (14) reported that although there were no observed differences in quality of life scores during reevaluation, 30% of patients with mild cognitive impairment or mild Alzheimer's disease and 40% of caregivers reported worsening of patients' health status during confinement.

### Caregivers

The pandemic and social isolation have also changed the lives of caregivers of older adults with dementia. Six studies assessed the pandemic's impact on caregivers (9, 16, 21, 27, 30). Only three of the six focused exclusively on caregivers (21, 27, 30). The others evaluated both caregivers and recipients of care. Two studies evaluated the caregivers qualitatively (28, 30), while the others used quantitative designs.

Increased burden of care, stress, and depressive symptoms had the most significant impact on caregivers. Altieri et al. (27) pointed to the association between resilience and symptoms of depression and anxiety. Caregivers with higher levels of resilience presented lower levels of depressive symptoms and high anxiety, and caregivers with low resilience showed an increase in anxiety symptoms alone. In addition, caregiver burden was associated with higher functional dependence. Vaitheswaran et al. (30) identified a two-way interaction between issues related to the caregiving role (protecting persons with dementia from infection or managing them when they were going to be hospitalized, isolated, or quarantined) and issues that were not related directly to their caregiving role (having to work from home due to lockdown). Additionally, caregivers suggested several methods such as video-consultations, telephone-based support, and in-person clinic-based visits to meet their needs (30). Boutoleau et al. (21) found that increased burden for caregivers of people with frontotemporal dementia (bvFTD) occurred regardless of behavioral changes, while caregivers of people with Alzheimer's disease experienced increased burden related to changes in the neuropsychiatric symptoms.

### Use of Technology

Technology has been an essential ally during the pandemic. All the selected studies performed data collection remotely. One study (29) compared the impact of additional services delivered either to care recipients and caregivers via video conference or to caregivers by telephone alone. They found varying degrees of improvements in physical and mental health, perceived burden, and self-efficacy in caregivers in the video-conferencing group that were absent in the telephone-only group (29). Goodman-Casanova et al. (13) reported that phone calls and video calls can offer social support and that some interventions can serve as recreational activities during the pandemic. Additionally, there were no significant differences in health and well-being between the intervention and control groups (13). Respondents with TV-AssistDem performed more memory exercises than control respondents. TV-AssistDem is a technological tool to facilitate remote support to people with mild cognitive impairment. It uses TV-based data transmission and video-interactivity between health professionals, patients, caregivers, and family members and provides such services as reminders, health monitoring, and cognitive stimulation (13).

Giebel et al. (12), in a longitudinal online or telephone survey, found that many older adults and people with dementia (PLWD) were less likely to be digitally literate, making it difficult for them to access services equally. A qualitative study by Portacolone et al. (15) found that some participants were satisfied with their telephone interactions with their physicians, but that digital illiteracy was a barrier to use of teleconferencing for others.

## Discussion

This systematic review aimed to elucidate the impact of the COVID-19 pandemic on people with dementia and on their caregivers' mental health. The database search yielded several articles related to the COVID-19 pandemic and dementia, but most of these studies addressed the mental health of health professionals, who are active on the front lines of the fight against the novel coronavirus. We also found studies on the mental health of older adults without neurocognitive disorders. Interestingly, there were few studies of people with dementia, possibly due to current limitations on research in this group. For example, standard neuropsychological assessment methods rely on face-to-face interactions, which were not possible due to social isolation. Social isolation requires modifications to study protocols for remote data collection to continue participants' assessments (6). We thus observed that many rating scales for measuring cognitive, behavioral, or mood symptoms in people with dementia were applied through videoconferencing or phone calls.

In most of the selected studies, caregivers helped people with dementia respond to the scales, a critical aspect of the assessments' reliability. Considering this study bias, Crivelli et al. (31) developed recommendations to support standardized clinical procedures that recommend data generation through teleneuropsychological assessments. For example, people with visual or auditory deficits, acute confusional states, or severe communication difficulties should not be evaluated using teleassessments, nor should they provide recorded verbal consent or an electronic signature. If tests are interrupted, they should be readministered from the beginning when contact with the patient is resumed, or it should be clarified that some qualitative data usually collected from face-to-face consultations are no longer acquired, which may limit recommendations and conclusions (31).

Concerning the studies' designs, we found only one longitudinal study. The cross-sectional studies used different methods of evaluation to assess the pandemic's impact. For example, Tsapanou et al. (16) provided a self-report questionnaire to caregivers of people with mild cognitive impairment or dementia related to changes in physical, psychological, and routine activities during the pandemic. Bouteleau-Bretonnière et al. (17) contacted caregivers of people with AD who were confined to their homes for nearly 2 months and asked about the changes in neuropsychiatric symptoms during this period. Mori et al. (24) compared the presence of depression in persons with Parkinson's disease and controls. Manini et al. (20) contacted caregivers of 109 community-dwelling adults with dementia who had a telephone follow-up after their hospital visits were canceled.

Interestingly, we found few studies evaluating cognition and level of functional impairment in people with dementia (9–11, 13, 17). Considering the different cognitive functions such as memory, attention, or executive function and their impact on different types of activities of daily living (basic and instrumental), it was not possible for the selected studies to examine which functions were most affected by social isolation. Therefore, further longitudinal studies should help to better understand the lockdown's impact on specific cognitive functions and routine activities to help develop interventions to attenuate the impact of social isolation on this population.

Social isolation is a measure to prevent spread of the novel coronavirus, but people with dementia and their caregivers have experienced changes in routine life, health services, and support activities as a result. Neuropsychiatric symptoms are a common feature in dementia, affecting 80% of patients over the course of the disease (6). Thus, most of the selected studies focused on changes in neuropsychiatric symptoms during social isolation. Overall, social isolation exacerbated or led to the manifestation of various neuropsychiatric symptoms. Agitation, delirium, irritability, apathy, aggression, anxiety, indifference, and altered mood were the most common symptoms found (9, 11, 14, 16, 18, 20, 21). Cohen et al. (18) found that new onset of behavioral symptoms or exacerbation of preexisting behavioral symptoms was positively correlated with patient's age and presence of anxiety reported before the epidemic and negatively correlated with global CDR score and the domains of memory, community affairs, and home and hobbies. Importantly, meaningful recreational activity has been shown to increase positive emotions, improve activities of daily living, and attenuate challenging neuropsychiatric symptoms (32). These findings may help develop potential digital delivery of non-pharmacological intervention programs, but further studies should explore differences in neuropsychiatric symptoms according to the type of dementia or patient's age at onset.

The COVID-19 pandemic has worsened the situation of families caring for people with dementia by delaying diagnosis and increasing the burden on caregivers (33). Caregivers have faced many challenges in caring for their loved ones, such as fear and concern about protecting them from SARS-CoV-2 infection, since recipients of care may not know how to follow the protective measures. Caregivers have had to reconcile the new challenges of care for their elders that already led to the burden of care (30). Furthermore, it is important to consider regional and cultural differences in caregiver support. For example, in Greece, Tsapanou et al. (16) reported that most families have lacked significant support during this period. In India, caregivers suggested several methods such as use of video-consultations, telephone-based support, and clinic-based in-person visits to meet their needs. They also requested more post-pandemic services (30). In Italy, caregivers with high resilience showed a more significant increase in anxiety levels during lockdown than caregivers with low resilience (27). There is thus a need to consider the development of specific interventions tailored to different cultural backgrounds and different types of dementia, such as early-onset dementia and more complex syndromes such as frontotemporal dementia (33).

Telemedicine has been a widely used method in this period. Lai et al. (29) studied whether telehealth would benefit people with dementia and their caregivers. The complementary telehealth delivered through video-conferencing apps was associated with more positive effects for community-dwelling older adults with neurocognitive impairment and their caregivers compared to conventional telehealth conducted by phone conversation only (29). In addition, there was a positive impact of telehealth via videoconferencing on cognition and a notable improvement in quality of life (29). One study (30) has suggested that online psychoeducational support and specific guidelines for care can meet caregivers' needs and contribute to their well-being. The use of technology by people with dementia and their caregivers depends on expectations, perceived skills, and expertise in using the devices (34). Some studies (12, 15) reported that many people with dementia were less likely to be digitally literate, making equal access to the services difficult. Therefore, technological initiatives should consider both people with cognitive impairment as well as different technology literacy levels.

## Future Directions

Another question is whether COVID-19 has a different clinical presentation in older people. Compared to younger people, the effect of COVID-19 on geriatric patients may be more serious because of higher rates of chronic illness, resulting in more severe cases of the disease. Unlike younger people, who present such symptoms as fever, cough, and chest discomfort, older adults may manifest COVID-19 through atypical symptoms such as mental confusion, falls, decreased mobility, tachycardia, blood pressure changes, decreased appetite, difficulty swallowing, and urinary incontinence (35). Therefore, caregivers of older adults may have difficulty recognizing the disease, especially in people with dementia, in whom these symptoms are already common. Older adults with dementia may also have other comorbidities that mask the infection. Prevention is still the safest measure against COVID-19, but screening services should consider that in older people, the infection can manifest itself through atypical symptoms. Thus, every geriatric patient should be tested and observed for all presenting symptoms (35). Medical teams and caregivers must be aware of any changes older adults may present, and hospitals must be prepared for the possible diagnosis. Misdiagnosis can lead to severe complications from the infection (36).

This systematic review has some limitations that should be considered. The first is the topic's broad scope, encompassing studies with multiple methods and outcomes. The second difficulty is transposing current evidence from one continent to another or from specific sociocultural and economic realities to others. The selected studies are also methodologically heterogeneous, thus limiting the comparison of their findings.

## Conclusions

There are few publications on the mental health of older persons with dementia and their caregivers during the COVID-19 pandemic compared to studies in other population groups. The selected studies were nearly unanimous in emphasizing that social isolation and withdrawal can lead to (or exacerbate) neuropsychiatric symptoms, motor difficulties, and cognitive decline. Caregivers have also suffered from the pandemic's impact, with an increase in the burden of care and symptoms of stress, depression, and anxiety. Both patients and caregivers have experienced radical changes in their routines that have affected their health and quality of life.

Few studies suggested measures to alleviate the difficulties of people with dementia and their caregivers. There are reports of the benefits of technology for communication and treatment via teleconsultations, but such technologies are still not widely known and not everyone has access to them, thus limiting their use by the target population. New forms of care and intervention are needed for older adults with cognitive impairment and their caregivers to prevent the intensification of their physical and psychological suffering.

## Data Availability Statement

The raw data supporting the conclusions of this article will be made available by the authors, without undue reservation.

## Author Contributions

JG performed the systematic search and wrote the first draft. IL assisted with articles inclusion and exclusion. MD designed the systematic review, assisted with articles inclusion and exclusion and approved the final version. All authors contributed to the article and approved the submitted version.

## Funding

MD receives research grants from the Brazilian National Council for Scientific and Technological Development (CNPq) and the Rio de Janeiro State Research Support Foundation (FAPERJ).

## Conflict of Interest

The authors declare that the research was conducted in the absence of any commercial or financial relationships that could be construed as a potential conflict of interest.

## Publisher's Note

All claims expressed in this article are solely those of the authors and do not necessarily represent those of their affiliated organizations, or those of the publisher, the editors and the reviewers. Any product that may be evaluated in this article, or claim that may be made by its manufacturer, is not guaranteed or endorsed by the publisher.
